# Effects of Xuesaitong on the Pharmacokinetics of Losartan: An *In Vivo* UPLC-MS/MS Study

**DOI:** 10.1155/2019/8373476

**Published:** 2019-08-14

**Authors:** Weina Ma, Lei Lv, Jungang Guo, Yongjun Meng, Yinghua Wang, Xiaoliang Yang, Lijun Zhu, Hua Nian, Liang Zhao

**Affiliations:** ^1^Department of Pharmacy, Jiading District Central Hospital Affiliated Shanghai University of Medicine & Health Sciences, Shanghai, China; ^2^Shanghai Eastern Hepatobiliary Surgery Hospital, Shanghai, China; ^3^Department of Thoracic Surgery, Jiading District Central Hospital Affiliated Shanghai University of Medicine & Health Sciences, Shanghai, China; ^4^Department of Pharmacy, Yueyang Hospital of Integrated Traditional Chinese Medicine and Western Medicine, Shanghai University of T.C.M., Shanghai, China; ^5^Department of Pharmacy, Shanghai Baoshan Luodian Hospital, Shanghai, China

## Abstract

The aim of this study was to examine whether Xuesaitong, a multiherbal formulation for coronary heart disease, alters the pharmacokinetics of losartan. Adult male Sprague Dawley rats randomly received losartan (10 mg/kg) or losartan plus Xuesaitong (10 mg/kg) through an oral gavage (*n* = 6). Multiple blood samples were obtained for up to 36 h to determine the concentrations of losartan and its active metabolite, EXP3174, through ultraperformance liquid chromatography-tandem mass spectrometry (UPLC-MS/MS). Pharmacokinetics were estimated using a noncompartmental model. The half-life (*t*_1/2_) of losartan was decreased by Xuesaitong (4.26 ± 1.51 vs. 6.35 ± 2.10 h; *P* < 0.05). The apparent volume of distribution (*V*_d_) of losartan was also decreased by the combination of losartan and Xuesaitong (4.41 ± 1.61 vs. 7.20 ± 2.41 mL; *P* < 0.05). The time to maximum concentration (*T*_max_) of losartan was increased by Xuesaitong (1.06 ± 1.04 vs. 0.13 ± 0.05 h; *P* < 0.05). Xuesaitong also decreased the *t*_1/2_ of EXP3174 (8.22 ± 1.41 vs. 6.29 ± 1.38 h; *P* < 0.05). These results suggest that there is a complex interaction between losartan and Xuesaitong. In addition to enhanced elimination of losartan and EXP3174, Xuesaitong may also decrease the absorption rate and *V*_d_ of losartan.

## 1. Introduction

Xuesaitong is a traditional Chinese medicine with multiple pharmacological activities. Major components of Xuesaitong are saponins from *Panax notoginseng* (PNS), including ginsenoside Rb_1_, ginsenoside Rg_1_, and notoginsenoside R_1_ [1]. *P*. *notoginseng* has been used to treat coronary artery disease for centuries in China [2, 3]. Xuesaitong increases blood supply to the myocardium, alleviates symptoms of angina pectoris, and reduces nitroglycerin dosage in patients with angina [4, 5]. Many patients receiving angiotensin-II-receptor antagonists for hypertension also receive Xuesaitong [6–8].

As a nonpeptide angiotensin-II-receptor antagonist, losartan is the first of its kind in the market [9–11]. Upon oral administration, losartan is metabolized into EXP3174—which is an active carboxylic-acid metabolite—by the cytochrome P450 subtypes, CYP3A4 and CYP2C9 [12, 13]. The elimination of EXP3174 also requires cytochromes CYP3A4 and CYP2C9 [12]. In comparison with losartan, EXP3174 has a much higher affinity for the angiotensin-II receptor and is mostly responsible for losartan's pharmacodynamic properties [14].

Metabolic drug interactions can occur when an herbal medicine and chemical pharmaceutical are coadministered such that the herbal medicine may alter the metabolism of the pharmaceutical via induction or obstruction of CYP450 isoforms [15]. A previous study showed that PNS can induce CYP1A2 [16], which ultimately affects the pharmacokinetic profiles of xenobiotics that are primarily metabolized by CYP1A2 [17]. A previous study in rats showed that notoginsenoside R_1_, a major active ingredient of Xuesaitong [18], inhibits CYP1A2 but not CYP2C11, CYP2D1, or CYP3A1/2 [19]. However, it is unclear whether other components in Xuesaitong affect the metabolism of losartan by CYP450 and whether some main components also influence the metabolism of losartan via CYP3A4 and CYP2C9. In this study, we examined the impact of Xuesaitong on the pharmacokinetic profile of losartan upon oral administration in rats. EXP3174 was examined simultaneously to capture the broader spectrum of potential interactions.

## 2. Methods

### 2.1. Materials and Reagents

**Figure 1:**
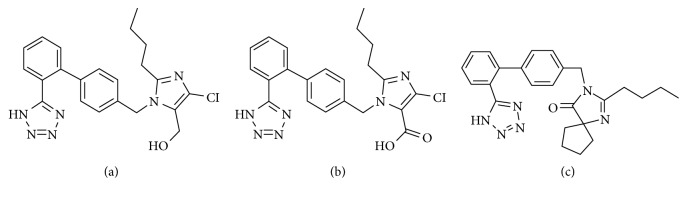
Structures of losartan (a), EXP3174 (b), and irbesartan (IS) (c).

Losartan (purity >98%) and irbesartan, the latter of which was used as an internal standard (IS; purity >98%), were obtained from the National Institute for the Control of Pharmaceutical and Biological Products (Beijing, China). EXP3174 (purity >98%) was obtained from Toronto Research Chemicals (Toronto, Canada).  Figure 1 shows the chemical structures of losartan, irbesartan, and EXP3174. Xuesaitong (tablet form) was purchased from Yunnanweihe Pharmaceutical Co., Ltd. (Yunnan, China). Losartan (tablet form) was purchased from Merck Sharp & Dohme Pty. Ltd. (South Granville, NSW, Australia). Formic acid and methanol were of mass-spectrometry grade and were acquired from Thermo Fisher Scientific (Waltham, MA, USA). A Milli-Q water-purification system (Millipore, Milford, MA, USA) was used to generate ultrapure water. Additional reagents were of analytical grade and were purchased from commercial companies.

### 2.2. Experimental Design

Approval was obtained from the Animal Ethics Committee of the Second Military Medical University (Shanghai, China) for all of the animal experimental procedures. Male adult Sprague Dawley rats (180–220 g; Sino-British SIPPR/BK Lab, Shanghai, China) were housed at 50 ± 10% relative humidity and 22 ± 2°C. Water and standard-rate chow were available *ad libitum*. The rats were allowed to acclimate for ten days and were then fasted for 12 h before experiments.

Twelve rats (six per group) randomly received losartan (10 mg/kg), either with or without Xuesaitong pretreatment (10 mg/kg, 5 min earlier), through an oral gavage. Dosage calculations were performed according to the body-surface-area ratio between human subjects and rats. A pestle was used to grind losartan and Xuesaitong tablets into powder, which was then dissolved in 5 mL of water before being added to a 10 mL of 1.5% Tween 80 solution (as a solubilizer) for homogenization. Blood samples (0.2 mL) were obtained via the tail vein at 0 (predosing), 5, 10, 15, and 30 min and 1, 2, 3, 4, 6, 8, 12, 24, and 36 h after the treatment. Plasma samples were stored at −80°C before being analyzed.

We used a noncompartmental model to obtain the following pharmacokinetic parameters with Phoenix WinNonlin 7.0 pharmacokinetic software (Pharsight Corporation, Princeton, NJ, USA): the area under the concentration-time curve from time 0 to the last-measured concentration (AUC_0–*t*_), the elimination half-life (*t*_1/2_), the area under the concentration-time curve from time 0 to infinity (AUC_0–inf_), the mean residence time (MRT), the area under the first-moment plasma concentration-time curve (AUMC), the apparent volume of distribution (*V*_d_), and the clearance rate (CL). Concentration-time curves were used to estimate the maximum plasma concentration (*C*_max_) and the time to reach the maximum concentration (*T*_max_). All of the results are presented as means ± standard deviations (SDs) and were compared with Student's *t-*tests by IBM SPSS 25.0 statistics software (IBM Corporation, Armonk, NY, USA). Statistical significance was determined at *P* < 0.05.

### 2.3. LC-MS/MS Parameters

An Agilent 1290 Infinity UPLC system (Agilent Technologies, USA) was used for chromatographic analysis and included a binary pump, a high-performance well-plate autosampler, an online vacuum degasser, and a temperature controller for the column. Samples were separated on a SHISEIDO MG-C_18_ column (100 mm × 3.0 mm, 3.0 *μ*m) and were eluted with an isocratic mobile phase of 0.1% formic acid-water and acetonitrile (50 : 50, v/v). The flow rate was 0.4 mL/min, the injection volume was 2 *μ*L, and the column temperature was 25°C. The analysis time was 5 min per specimen.

**Figure 2:**
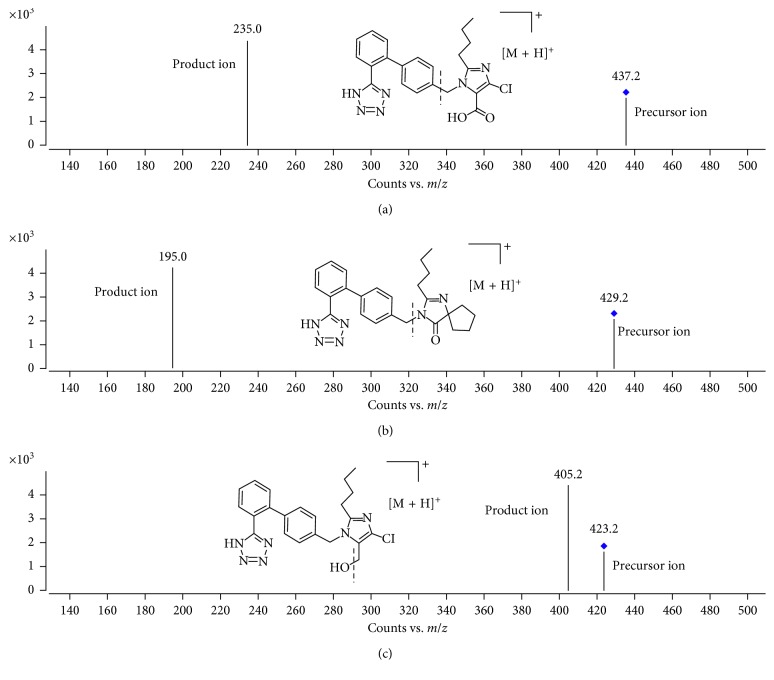
Precursor and product ions of EXP3174 (a), irbesartan (IS) (b), and losartan (c).

The LC system was linked to an Agilent 6470 Triple Quad MS system (Agilent Technologies, USA) with an Agilent Jet Stream Technology (AJS) electrospray-source interface (ESI). The positive-ion mode was optimized for MS detection via multiple-reaction monitoring (MRM). Precursor and product ions had *m*/*z* 423.2 ⟶ 405.2 for losartan, *m*/*z* 429.2 ⟶ 195 for IS, and *m*/*z* 437.2 ⟶ 235 for EXP3174. The precursor and product ions of losartan, EXP3174, and IS are shown in Figure 2; the collision energies for each were 10, 20, and 20 eV, respectively. The fragmentor voltage for each was 100 V. The ionized chamber-working parameters were optimized as follows: the capillary voltage was 4000 V, drying gas flow was 10 L/min, gas temperature was 350°C, nebulizer pressure was 40 psi, sheath gas temperature was 400°C, and sheath gas flow was 11 L/min. Agilent MassHunter Workstation version B.07.00 was used to acquire and analyze the data.

### 2.4. Quality Control Samples and Calibration Standards

Losartan (1.0 mg/mL) and EXP3174 (1.0 mg/mL) stock solutions were prepared using reference standards in methanol solution. The stock solution of IS was diluted to 100 ng/mL prior to use. Working standard solutions of losartan and EXP3174 were prepared by combining and diluting each stock solution with methanol. Additional standard working solutions were prepared by dilution with methanol. The working solutions were spiked into blank rat plasma at concentrations of 2–2000 ng/mL (2, 5, 10, 50, 100, 500, 1000, 1600, and 2000 ng/mL) to establish calibration standards for losartan and EXP3174. Quality control (QC) samples for validation were similarly set at 5, 100, and 1600 ng/mL. Standards and QC specimens were removed on every analysis day via a method identical to that used for actual samples, as detailed below.

### 2.5. Plasma Sample Preparation

Portions of 50 *μ*L of plasma, 50 *μ*L of methanol, and 100 *μ*L of IS (100 ng/mL) were combined via 30 s of vortexing; this combination was then centrifuged at 13,800 *g* for 5 min. Supernatants were transferred to injection vials, and an aliquot of 2 *μ*L was analyzed by UPLC-MS/MS.

### 2.6. Method Validation

We evaluated method selectivity by examining six separate blank rat-plasma samples that we contrasted with the ones acquired by placing analytes into correlating blank plasma specimens with IS to observe interference. Calibration curves for losartan and EXP3174 comprised plots of the peak-area ratio of the analyte to IS against plasma concentration with a 1/*x* weighting. Least-squares linear regression was used to establish the intercept, slope, and correlation coefficient of the equation. The lower limit of quantification (LLOQ) was defined as the analyte concentration with a signal-to-noise ratio of 10.

The accuracy and precision for both interday and intraday were determined with QC specimens for three consecutive days at three concentrations. Five replicates were used for each concentration. Each analyte concentration was quantified with a calibration curve on the same testing day.

Analyte-extraction recovery was performed by contrasting the peak areas acquired from blank plasma, in which analytes had been combined prior to removal, with areas from samples, in which they were combined following removal; this was performed five times. The matrix effect was examined by contrasting the solution in which the blank processed matrix had been combined with the solution at various QC concentrations; this procedure was performed five times.

To determine the stability of samples, three QC samples at high, medium, and low concentrations were examined under various conditions, including short-term storage stability at room temperature for 3 h, postpreparative stability with the autosampler for 24 h, three freeze-thaw cycles at −80°C, and long-term storage stability at −80°C for 30 days.

## 3. Results

### 3.1. Method Validation

#### 3.1.1. Specificity

**Figure 3:**
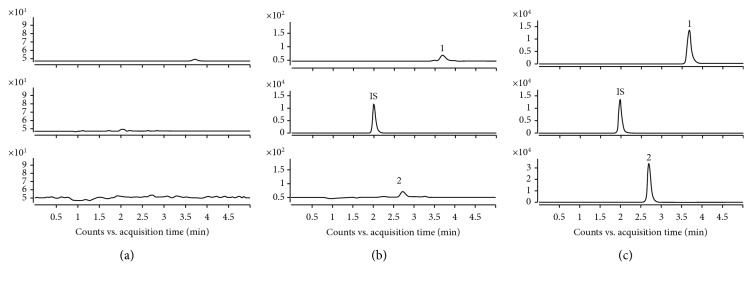
Chromatograms of (a) blank plasma; (b) blank plasma with losartan, EXP3174, and irbesartan (IS); and (c) rat plasma after oral administration. In the chromatograms, “1” denotes EXP3174, “2” denotes losartan, and “IS” denotes irbesartan.

Figure 3 shows LC-MS/MS chromatograms that are representative of losartan, IS, and EXP3174. The retention time was 2.7 min for losartan, 2.1 min for IS, and 3.7 min for EXP3174. No significant endogenous elements obstructed the analytes or IS.

#### 3.1.2. LLOQ and Calibration Curves

The LLOQ for losartan and EXP3174 with S/N >10 was 0.5 ng/mL and 1.0 ng/mL, respectively. Calibration curves for losartan and EXP3174 were created by plotting the peak-area ratios of the analytes to IS against the plasma concentration with a 1/*x* weighting. The calibration curves for losartan and EXP3174 were *y* = 0.0042*x* + 0.0057 (*r* = 0.996) and *y* = 0.0018*x* + 0.0019 (*r* = 0.999), respectively.

#### 3.1.3. Precision and Accuracy

Five separate runs on the same day for five consecutive days with three QC sample concentrations were performed for evaluating interday and intraday comparisons. The precision (relative standard deviation (RSD)) of this technique was <7% for losartan and EXP3174. The accuracy (relative error (RE)) was from −9.78% to 7.78% for losartan and −9.92% to 3.06% for EXP3174. The results are indicated in Table 1.

#### 3.1.4. Extraction Recovery and Matrix Effect

The extraction recoveries for losartan and EXP3174 were >85%, and there were no significant variations among the three concentrations. The analyte-matrix effect ranged from 92.43 to 115.74%. The results are indicated in Table 2.

#### 3.1.5. Stability

We evaluated stability using a variety of conditions. Both losartan and EXP3174 remained stable at 4°C for 3 h at room temperature, for 24 h in the autosampler after three cycles of freeze-thawing, and after long-term storage for 30 days at −80°C. The concentration variation was <10%. The results are indicated in Table 3.

#### 3.1.6. Carryover and Dilution

No detectable carryover was noted. The accuracy was within ±15.0%, and the precision was under 15%, according to dilution-integrity experiments.

### 3.2. In Vivo Pharmacokinetic Study

**Figure 4:**
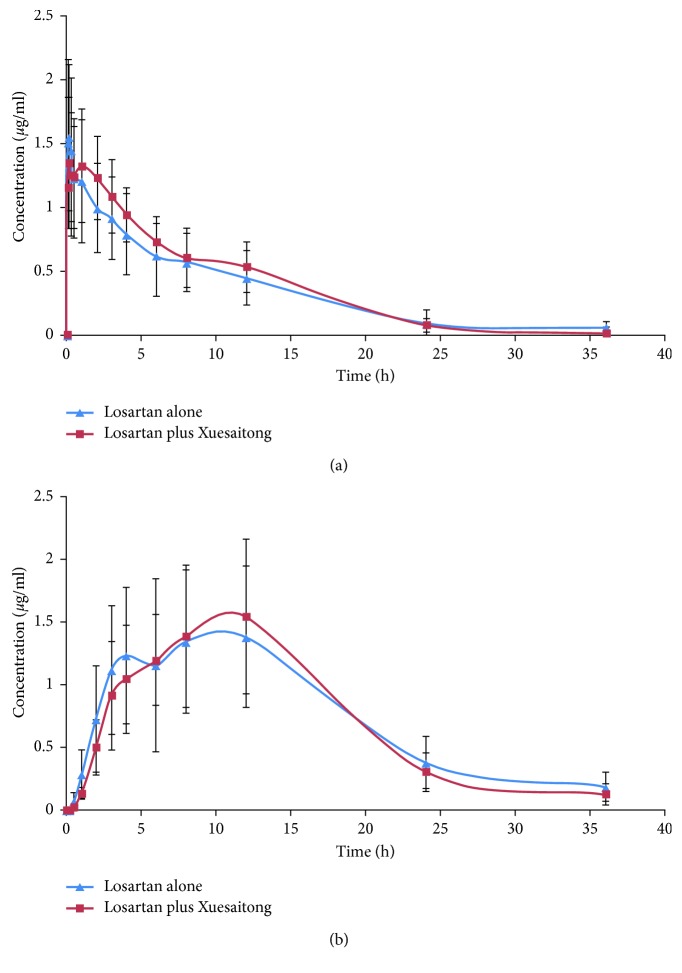
Mean concentration-time curves upon losartan treatment vs. losartan plus Xuesaitong. (a) Losartan. (b) EXP3174. The error bars represent standard deviations (SDs).

Concentration-time curves for losartan and EXP3174 in rat plasma (upon losartan treatment vs. losartan plus Xuesaitong treatment) are shown in Figure 4. Pharmacokinetic parameters were determined with the WinNonlin 7.0 pharmacokinetic program based on a noncompartmental pharmacokinetic model. As indicated in Table 4, the *t*_1/2_, *V*_d_, and *T*_max_ of losartan were 4.26 ± 1.51 h, 4.41 ± 1.61 mL, and 1.06 ± 1.04 h in rats receiving losartan plus Xuesaitong vs. 6.35 ± 2.10 h, 7.20 ± 2.41 mL, and 0.13 ± 0.05 h in rats receiving losartan alone, respectively; these results indicated that Xuesaitong decreased the *t*_1/2_ and *V*_d_ of losartan and increased the *T*_max_ (*P* < 0.05 for all). The *t*_1/2_ of EXP3174 was 6.29 ± 1.38 h in rats receiving losartan plus Xuesaitong vs. 8.22 ± 1.41 h in rats receiving losartan alone, indicating that Xuesaitong decreased the *t*_1/2_ of EXP3174 (*P* < 0.05).

## 4. Discussion

### 4.1. Method Development

To optimize the pretreatment, we chose the simple method of protein precipitation and studied the effects of precipitation of methanol and acetonitrile as well as the influence of volume ratio on the effects of precipitation. The results showed that methanol protein deposition using the same volume as the plasma yielded the most optimal results. The extraction efficiency surpassed 90% with methanol as the extraction solution. The direct precipitation method also revealed convenience with the low matrix effect, indicating that it was an excellent precipitating agent.

To determine the suitable retention time and reaction of losartan and EXP3174 in rat plasma, water, methanol, formic acid, and acetonitrile were examined as mobile phases. Solvent A (0.1% formic acid) and solvent B (acetonitrile; 50 : 50, v/v) after optimization were determined to improve the ionization efficiency and led to an elevated intensity compared to water for each constituent evaluated. To devise a precise and sensitive LC-MS/MS method, we conducted quantitative analysis in the MRM mode because of its high sensitivity and selectivity. Precursor and product ions had *m*/*z* 423.2 ⟶ 405.2 for losartan, *m*/*z* 429.2 ⟶ 195 for IS, and *m*/*z* 437.2 ⟶ 235 for EXP3174. We optimized MS/MS conditions with an optimization program to improve sensitivity and selectivity. The technique could be simply reproduced, and it was fully validated and successfully used in the pharmacokinetics evaluation in rats after orally administering losartan and Xuesaitong.

### 4.2. Losartan/Xuesaitong Interactions

According to the results shown in Table 4, changes in *t*_1/2_ suggest that Xuesaitong could shorten the elimination time of losartan and EXP3174, increase losartan metabolism, and reduce the therapeutic effect of losartan. Changes in *V*_d_ suggest that Xuesaitong could decrease the distribution of losartan and induce its metabolism. Changes in *T*_max_ suggest that Xuesaitong could decrease the absorption rate of losartan. These results suggest that the losartan dosage should be increased when Xuesaitong is concurrently used.

Interactions between losartan and Xuesaitong could occur during absorption, metabolism, distribution, and/or excretion. A previous study indicated that tanshinone IIA and salvianolic acid B (two components of danshen) have distinct effects on the metabolism of losartan. Tanshinone IIA inhibits losartan metabolism via CYP3A4 and CYP2C9; salvianolic acid B, in contrast, induces losartan metabolism via CYP3A4 and CYP2C9 [20]. Losartan is mainly metabolized by CYP450 [21]. In view of the previous studies, we speculate that Xuesaitong may alter losartan and EXP3174 pharmacokinetics by regulating CYP3A4 and CYP2C9 expression and/or activities. The current results suggest that patients who take Xuesaitong and losartan concomitantly should increase their dosage of losartan.

## 5. Conclusions

In this study, we developed a UPLC-MS/MS technique with high sensitivity and accuracy to measure losartan and EXP3174 in rat plasma. We used this technique successfully for the pharmacokinetic evaluation of losartan and EXP3174 following losartan and Xuesaitong oral administration. Our results showed that Xuesaitong affected the pharmacokinetics of losartan following coadministration. We recommend an increased dosage of losartan in patients who also receive Xuesaitong.

## Figures and Tables

**Table 1:** Accuracy and precision of losartan and EXP3174 in rat plasma.

Compounds	Concentration (ng·mL^−1^)	Intraday (*n* = 5)		Interday (*n* = 15)	
		RSD (%)	RE (%)	RSD (%)	RE (%)
Losartan	5	1.19	−9.78	4.03	−8.45
	100	1.52	4.90	1.07	4.60
	1600	2.00	7.78	2.58	6.01
EXP3174	5	5.89	−9.92	6.46	−8.77
	100	2.22	2.65	4.68	−0.53
	1600	3.62	3.06	4.30	0.83

RSD, relative standard deviation; RE, relative error.

**Table 2:** Matrix effect and extraction recovery of losartan and EXP3174 in rat plasma (*n* = 6).

Compounds	Concentration (ng·mL^−1^)	Matrix effect (%)	RSD (%)	Recovery (%)	RSD (%)
Losartan	5	92.43	8.69	85.60	6.34
	100	93.25	4.04	108.28	1.46
	1600	96.82	1.34	111.12	0.96
EXP3174	5	101.98	6.27	85.19	2.71
	100	115.74	7.75	101.25	2.38
	1600	111.36	2.59	109.79	2.51

RSD, relative standard deviation; RE, relative error.

**Table 3:** Stability outcomes of losartan and EXP3174 in rat plasma (*n* = 5).

Compounds	Concentration (ng·mL^−1^)	Storage at 25°C for 3 h		Storage at 4°C for 24 h		Three freeze-thaw cycles		Long-term storage at −80°C for 30 days	
		Measured (ng·mL^−1^)	RE (%)	Measured (ng·mL^−1^)	RE (%)	Measured (ng·mL^−1^)	RE (%)	Measured (ng·mL^−1^)	RE (%)
Losartan	5	5.25	5.00	5.47	8.30	5.39	6.71	5.41	8.31
	100	104.60	4.60	109.52	9.52	106.54	6.54	107.46	7.46
	1600	1633.48	2.09	1673.44	4.59	1576.38	−1.48	1686.27	5.39
EXP3174	5	5.37	7.40	5.49	7.76	5.44	7.78	5.46	7.15
	100	102.33	2.33	107.57	7.57	97.22	−2.78	94.40	−5.60
	1600	1648.96	3.06	1653.63	3.35	1487.01	−7.06	1492.90	−6.94

RSD, relative standard deviation; RE, relative error.

**Table 4:** Pharmacokinetic parameters of losartan and EXP3174 in rats following oral administration of two pretreatments (A: losartan alone; B: losartan plus Xuesaitong).

Parameter	Losartan		EXP3174	
	Losartan alone (A)	Losartan plus Xuesaitong (B)	Losartan alone (A)	Losartan plus Xuesaitong (B)
*t*_1/2_ (h)	6.35 ± 2.10	4.26 ± 1.51^*∗*^	8.22 ± 1.41	6.29 ± 1.38^*∗*^
*T*_max_ (h)	0.13 ± 0.05	1.06 ± 1.04^*∗*^	6.83 ± 4.12	11.33 ± 1.63^*∗*^
*C*_max_ (*μ*g·mL^−1^)	1.60 ± 0.59	1.50 ± 0.49	1.61 ± 0.61	1.60 ± 0.54
AUC_0–*t*_ (*μ*g·mL^−1^·h)	12.92 ± 5.01	14.17 ± 4.21	27.02 ± 10.49	26.40 ± 10.08
AUC_0–inf_ (*μ*g·mL^−1^·h)	13.54 ± 5.54	14.28 ± 4.23	29.40 ± 11.85	27.64 ± 10.88
AUMC_0–*t*_ (*μ*g·mL^−1^·h^2^)	101.66 ± 26.75	120.88 ± 27.41	324.38 ± 65.68	320.31 ± 58.17
AUMC_0–inf_ (*μ*g·mL^−1^·h^2^)	101.15 ± 26.13	127.30 ± 25.52	440.49 ± 34.34	361.21 ± 36.31
MRT_0–*t*_ (h)	8.41 ± 1.80	7.76 ± 0.88	12.42 ± 1.48	11.98 ± 1.27
MRT_0–inf_ (h)	9.86 ± 2.80	8.03 ± 1.00	15.09 ± 2.72	13.31 ± 2.08
*V*_d_ (mL)	7.20 ± 2.41	4.41 ± 1.61^*∗*^	4.52 ± 1.66	3.62 ± 1.21
CL (mL·h^−1^)	0.84 ± 0.32	0.77 ± 0.31	0.39 ± 0.16	0.42 ± 0.21

^*∗*^ *P* < 0.05 (losartan plus Xuesaitong vs. losartan alone).

## Data Availability

The raw/processed data required to reproduce these findings are available from the corresponding author upon request.

## Abbreviations

AJS: Agilent Jet Stream Technology
AUC: The area under the concentration-time curve
AUMC: The area under the first-moment plasma concentration-time curve
CL: The clearance rate
*C*_max_: The maximum plasma concentration
*V*_d_: The apparent volume of distribution
ESI: Electrospray-source interface
IS: Internal standard
LLOQ: Lower limit of quantification
MRM: Multiple-reaction monitoring
MRT: The mean residence time
PNS: *Panax notoginseng* saponins
QC: Quality control
RE: Relative error
RSD: Relative standard deviation
SD: Standard deviation
*t*_1/2_: The elimination half-life
*T*_max_: The time to reach the maximum concentration
UPLC-MS/MS: Ultraperformance liquid chromatography-tandem mass spectrometry.
